# Protocol for Cancloz: multicentre randomised, placebo-controlled, double-blind, parallel-group adaptive trial of cannabidiol for clozapine-resistant schizophrenia

**DOI:** 10.1192/bjo.2024.748

**Published:** 2024-10-03

**Authors:** Dan Siskind, Claudia Bull, Shuichi Suetani, Nicola Warren, Anastasia Suraev, Iain McGregor, Steve Kisely, Veronica De Monte, Mike Trott, Manju Shine, Vikas Moudgil, Gail Robinson, Stephen Parker, Ravikumar Krishnaiah, Terry Stedman, Allan Drummond, Sarah Medland, Ravi Iyer, Andrea Baker

**Affiliations:** Faculty of Medicine, The University of Queensland, Woolloongabba, Australia; Metro South Addiction and Mental Health Services, Woolloongabba, Australia; Queensland Centre for Mental Health Research, Wacol, Australia; and Queensland Brain Institute, The University of Queensland, Brisbane, Australia; Faculty of Medicine, The University of Queensland, Woolloongabba, Australia; and Queensland Centre for Mental Health Research, Wacol, Australia; Queensland Centre for Mental Health Research, Wacol, Australia; Queensland Brain Institute, The University of Queensland, Brisbane, Australia; Institute for Urban Indigenous Health, Windsor, Australia; and School of Medicine and Dentistry, Griffith University, Southport, Australia; Faculty of Medicine, The University of Queensland, Woolloongabba, Australia; Metro South Addiction and Mental Health Services, Woolloongabba, Australia; and Queensland Centre for Mental Health Research, Wacol, Australia; Lambert Initiative for Cannabinoid Therapeutics, University of Sydney, Sydney, Australia; Faculty of Medicine, The University of Queensland, Woolloongabba, Australia; and Metro South Addiction and Mental Health Services, Woolloongabba, Australia; Metro South Addiction and Mental Health Services, Woolloongabba, Australia; Metro North Mental Health, Royal Brisbane Women's Hospital, Herston, Australia; Metro North Mental Health, The Prince Charles Hospital, Chermside, Australia; Faculty of Medicine, The University of Queensland, Woolloongabba, Australia; School of Medicine and Dentistry, Griffith University, Southport, Australia; and Metro North Mental Health, Royal Brisbane Women's Hospital, Herston, Australia; Community Mental Health, Gold Coast University Hospital, Southport, Australia; West Moreton Division of Mental Health and Specialised Services, Wacol, Australia; Goodna Community Mental Health, Goodna, Australia; and Integrated Mental Health Centre, Ipswich, Australia; QIMR Berghofer Medical Research Institute, Herston, Australia; MAGNET: Mental Health Australia General Clinical Trials Network, Geelong, Australia; and Swinburne University of Technology, Hawthorn, Australia; Queensland Centre for Mental Health Research, Wacol, Australia

**Keywords:** Schizophrenia, cannabidiol, clozapine, treatment-resistant schizophrenia, randomised controlled trial

## Abstract

**Background:**

Although clozapine is the most effective antipsychotic for people with treatment-resistant schizophrenia (TRS), only 40% of people with TRS respond, and there is limited evidence for augmentation agents. Cannabidiol (CBD) reduces positive symptoms in individuals with schizophrenia, but no trials have specifically examined its efficacy in those with clozapine-resistant schizophrenia.

**Aims:**

To examine the clinical efficacy of CBD augmentation in people with clozapine-resistant schizophrenia.

**Method:**

This is a 12-week randomised, placebo-controlled, double-blind, parallel-group trial (registration number: ACTRN12622001112752). We will recruit 88 individuals with clozapine-resistant schizophrenia, randomised (1:1) to 1000 mg daily CBD versus placebo. Eligible individuals will be aged between 18 and 64 years, fulfil DSM-IV criteria for schizophrenia or schizoaffective disorder, have a total PANSS (Positive and Negative Syndrome Scale) score ≥60, have received oral clozapine for at least 18 weeks and have a clozapine level of >350 ng/mL. Interim analyses will be conducted at 25, 50 and 75% recruitment; these will also provide an opportunity to reallocate participants dependent on conditional power. The primary endpoint will be the difference in PANSS positive scores at the end of week 12. Secondary endpoints include depression, anxiety, sleep, quality of life, alcohol consumption, change in weight and metabolic syndrome components, and neurocognitive measures, as well as safety and tolerability.

**Discussion:**

Novel treatments for clozapine-resistant schizophrenia are urgently needed. If found to be effective, CBD may have a role as a novel and safe adjunct to clozapine.

Schizophrenia, which is characterised by disturbances in cognition, affect, perception and behaviours, affects approximately 1% of the population;^1^ however, schizophrenia can be difficult to treat, even with optimal therapy. Roughly one-third of people with schizophrenia have treatment-resistant schizophrenia (TRS),^2^ defined as ongoing symptoms and functional impairment despite two adequate and adherent trials of different antipsychotics.^3^

Although antipsychotic medications and supportive therapies reduce symptom burden and relapse rate, many individuals with schizophrenia continue to have ongoing symptoms and concomitant cognitive and social disabilities, poor physical health and curtailed life expectancy.^4^ Currently, the most effective antipsychotic for TRS is clozapine, which leads to reductions in positive symptoms and hospital admissions.^5,6^ Even so, only 40% of people with TRS trialled on clozapine meet clinical response criteria.^7^ For people with clozapine-resistant schizophrenia, there are few agents available to augment treatment, and these have limited effectiveness.^8^

The endocannabinoid system has a strong neuromodulatory role, with effects on inflammation, cell proliferation and apoptosis, pain modulation, memory and learning, and fear, among other functions.^9^ Endocannabinoids bind to presynaptic receptors, including CB1 and CB2, to potentiate retrograde signals that regulate the release of other neurotransmitters, such as dopaminergic, serotonergic, adrenergic, cholinergic and γ-aminobutyric acid (GABA).^10,11^ Cannabidiol (CBD) is the non-intoxicating component of the cannabis plant. Compared with delta-9-tetrahydrocannabinol (THC), CBD has a more anxiolytic profile, sparing disturbances in perception and thought processing.^12^ CBD has strong potential for neuropsychiatric therapeutic use, as it acts on the central nervous system via neuroreceptors including CB1, the serotonin 5-HT1A receptor and the transient receptor potential vanilloid type 1 receptor.^13^ Recent studies have explored the potential of CBD to target the endocannabinoid system to improve mood and anxiety, epilepsy, chronic pain, movement disorders (such as Parkinson's disease), nausea and inflammation associated with metabolic syndrome, malignancy, atherosclerosis, etc.^11,13^ Following animal and *in vivo* studies, it has been suggested that CBD is non-toxic, with minimal (usually mild) side-effects.^14^ CBD has shown promise as a treatment for mental disorders, including anxiety, insomnia and post-traumatic stress disorder.^15^

There have been three randomised placebo-controlled trials, albeit not among people with clozapine-resistant schizophrenia.^16–18^ A 6-week double-blind parallel-group trial of 88 participants with partially responsive schizophrenia, not trialled on clozapine, compared adjunctive CBD 1000 mg/day with placebo.^17^ This study found significantly greater improvements in positive psychotic symptoms in the CBD group. However, it failed to reach significance with respect to the difference in the proportions of participants in the two groups showing ≥20% improvement in PANSS (Positive and Negative Syndrome Scale) total score. A second 4-week blind randomised controlled trial (RCT) among 42 participants receiving 600–800 mg CBD daily versus the standard antipsychotic medication, amisulpride, showed improvements in psychotic symptoms with both treatments and fewer adverse drug reactions in the CBD arm.^18^ However, the third study, a double-blind 6-week randomised controlled trial in 36 treatment-responsive patients with schizophrenia did not find a difference in psychotic symptoms between groups receiving adjunctive CBD 600 mg/day and placebo.^16^ Other non-randomised or single-dose CBD studies have shown equivocal results.^19^

CBD has been found to be well tolerated at different doses across several clinical trials. A trial by Taylor et al in healthy controls showed good tolerance of single CBD doses of 1500, 3000, 4500 and 6000 mg.^20^ In patients receiving multiple doses (750 and 1500 mg twice daily) for 6 days, CBD was also well tolerated, with no participants leaving the trial owing to adverse events.^20^ McGuire et al trialled doses of 1000 mg daily of CBD in patients with schizophrenia in addition to their usual antipsychotic medications for 6 weeks.^17^ Positive psychotic symptoms were lower in the treatment group, with similar adverse events to those of the placebo group, showing good tolerance for CBD.^17^

To date, there have been no trials of CBD among people with clozapine-resistant schizophrenia. This trial has the potential to identify CBD – if found to be effective – as a novel and safe adjunctive treatment for individuals with clozapine-resistant schizophrenia.

## Study objectives

The aim of this study is to examine the clinical efficacy of an add-on CBD treatment in patients with clozapine-resistant schizophrenia. The primary objective is to determine the impact of 12-week treatment with CBD, compared with placebo, on scores on the positive subscale of PANSS in patients with clozapine-resistant schizophrenia.

## Hypothesis

We hypothesise that participants allocated to the intervention group (1000 mg daily CBD treatment) will demonstrate a reduction in PANSS positive scores at the end of week 12 compared with participants receiving placebo.

## Method

### Design and setting

The study is a randomised, placebo-controlled, double-blind parallel-group adaptive trial to examine the clinical efficacy and safety of add-on CBD treatment in patients with clozapine-resistant schizophrenia. It will recruit 88 individuals with clozapine-resistant schizophrenia, who will be randomised at the point of providing written informed consent. The study design was discussed with our local lived experience reference group.

Participants will be given 1000 mg daily of CBD or placebo, in addition to routine care. A battery of validated clinical and physical health measures will be conducted throughout the trial period. Adverse events will be recorded fortnightly (i.e. weeks 0, 2, 4, 6, 8, 10 and 12), and trial medication will be distributed to participants fortnightly (i.e. weeks 0, 2, 4, 6, 8 and 10).

The authors assert that this study complies with the ethical standards of the relevant national and institutional committees on human experimentation and with the World Medical Association Declaration of Helsinki *Ethical Principles for Medical Research Involving Human Subjects*.^21^ All procedures involving human participants were approved by the Metro South Human Research Ethics Committee (reference number: HREC/2022/QMS/83530). The study sponsor is the University of Queensland, which provides overarching governance. We have separate site governance approvals for each recruiting site. The trial is registered on the Australia New Zealand Clinical Trials Registry (registration number: ACTRN12622001112752). The study will follow the CONSORT guidelines for parallel-group randomised trials.^22^

### Participants

Eighty-eight eligible participants with TRS will be recruited through mental health services at the Gold Coast, Metro North, Metro South, and West Moreton Hospital and Health Services and in south-east Queensland, Australia. Participant eligibility is detailed in Box 1. Participants must meet all the inclusion criteria and none of the exclusion criteria. Capacity to consent will be determined by the treating clinician in collaboration with clinical trial staff. Only participants with this capacity will be recruited following their provision of consent.

Box 1 Participant eligibility

**Table:** 

Inclusion criteria
Individuals will be invited to participate in the study if they meet all the following criteria:
(a) aged between 18 and 64 years (inclusive);
(b) meet DSM-IV criteria for schizophrenia or schizoaffective disorder, based on the Diagnostic Interview for Psychoses^23^;
(c) have clozapine-resistant schizophrenia as defined by total Positive and Negative Syndrome Scale score ≥60;
(d) have received oral clozapine for a period of ≥18 weeks, with a clozapine level of >350 ng/mL;
(e) agree to participate in the trial, have the capacity to consent, and can follow study instructions and procedures.
Exclusion criteria
Individuals will not be invited to participate in the study if they meet any of the following criteria:
(a) are currently pregnant, lactating, or not using effective contraception if sexually active (applicable to both male and female patients);
(b) present clinical blood test results that might compromise patient safety or confound trial results;
(c) are currently using psychotogenic substances including amphetamine and cannabis (by self report);
(d) are actively using other prescribed cannabinoids;
(e) are experiencing severe disturbance, such that the person is unable to comply with either the requirements of informed consent or the treatment protocol;
(f) have any concomitant disease or condition that according to the investigator's assessment makes the patient unsuitable for trial participation;
(g) have ceased using clozapine.

### Allocation concealment and randomisation

Participants will be randomised using a chronological process and only after written consent has been provided and baseline assessments have been completed. Participants will be randomised to either the intervention or control group in a 1:1 ratio using a computer-generated randomisation table with block randomisation. They will be allocated a unique identification number, which will be linked to a specific number based on the site they have been recruited from. If a participant withdraws from the study, their identification number will not be re-used, nor will they be allowed to re-enter the study.

Participants, clinical trial staff, investigators and treating clinicians will be blinded to the intervention. An independent biostatistician will generate the randomisation list, which will be provided to the manufacturer and a study site pharmacist. The pharmacist will hold the closed randomisation list and will be the only one who is able to unblind participants. In the case of an emergency, where it is critical that medical staff know whether a participant is on CBD or placebo, participants will be provided with contact information for unblinding (i.e. a 24-h number).

### Pharmacological treatment

Participants will receive five gel capsules per day containing either 200 mg CBD (99% purity; <0.1 mg THC per capsule) suspended in medium-chain triglyceride oil or matched placebo (medium-chain triglyceride oil only). Capsules were manufactured by Linnea SA, Lavertezzo, Switzerland, a Therapeutic Goods Administration licensed facility, in accordance with current Good Manufacturing Practice and distributed by BOD Science. Dispensation, by a clinical trial staff member, will occur on a fortnightly basis, with six dispensations total per participant.

### Outcomes

#### Primary

The primary outcome was the impact of 1000 mg daily CBD treatment over 12 weeks compared with placebo on PANSS positive score in participants with clozapine-resistant schizophrenia.

#### Secondary

Secondary outcomes included the impact of 1000 mg daily CBD treatment over 12 weeks compared with placebo on PANSS total scores and other PANSS subscales; depression; anxiety; sleep; quality of life; alcohol consumption; changes in weight and metabolic syndrome components including waist circumference, haemoglobin A1C (HbA1c), high-density lipoprotein (HDL), low-density lipoprotein (LDL), body mass index (BMI), triglycerides, blood pressure, hip-to-waist ratio, electrolyte and liver function tests (ELFTs), non-alcoholic fatty liver disease (NAFLD) scores, fibrosis-4 scores (FIB-4), heart rate, diet and appetite, and physical activity; endpoint clozapine and norclozapine levels compared with baseline; and neurocognitive measures. Additional aims were to determine the safety and tolerability over 12 weeks of 1000 mg daily CBD in patients with clozapine-resistant schizophrenia compared with placebo as measured by the number of participants reported to have dropped out, number of adverse events reported, and scores from a structured qualitative interview with participants about their experience with the study investigational product using the Systematic Assessment for Treatment Emergent Events – Systematic Inquiry (SAFTEE-SI).^24^

#### Tertiary

Tertiary outcomes were changes between baseline and the 12-week endpoint in CBD and CBD metabolites (7-COOH-CBD, 6-OH-CBD, 7-OH-CBD), medication adherence, and measurements of THC and its metabolites (11-COOH-THC, 11-OH-THC) to check for possible illicit cannabis use.

### Measurements

Table 1 illustrates the trial schedule of visits and assessments. A range of validated clinical and physical health measures will be administered at different points throughout the trial period. Clinical measures will be administered by clinical trial staff, whom the clinical trial research manager will supervise. These staff are experienced mental health clinicians trained to administer these measures. Depending on participants' preferences, clinical measures will be administered in the clinic or at the participant's residence.

**Table 1:** Schedule of trial visits and assessments

Visit	0 (Screening)	1 (Baseline)	2	3	4	5	6	7
							
Week		0 (Baseline)	2	4	6	8	10	12
Study medication period (12 weeks)								
Screening and consent								
Assessment of current medication	x	x	x	x	x	x	x	x
Informed consent	x							
Ongoing capacity	x	x	x	x	x	x	x	x
Inclusion/exclusion criteria	x							
Urine pregnancy test (females only)	x							
Investigational product supply (fortnightly)		x	x	x	x	x	x	
Safety^a^								
Dropouts								x
Adverse events		x	x	x	x	x	x	x
SAFTEE-SI		x	x	x	x	x	x	x
Efficacy^a^								
Height		x						
Body weight		x		x		x		x
Waist circumference, waist-to-hip ratio		x		x		x		x
Heart rate		x		x		x		x
Blood pressure		x		x		x		x
HDL, LDL, triglycerides, total cholesterol		x						x
HbA1c		x						x
NAFLD, FIB-4		x						x
PANSS total and subscale scores		x		x		x		x
GAF		x						x
SIMPAQ		x						x
AQoL-4D		x						x
CGI		x						x
TOPF		x						x
BACS verbal memory and learning		x						x
BACS digit sequencing task		x						x
BACS symbol coding task		x						x
BASC semantic and letter fluency		x						x
HRSA		x						x
CDSS		x						x
TMT		x						x
FCI		x						x
AUDIT		x						x
PSQI		x						x
Other^a^								
Trial medication compliance			x	x	x	x	x	x
Blood (other) – FBC (WCC, Neutrophils, lymphocytes), ELFT, clozapine/ norclozapine levels		x			x			x
Blood (other) – CBD and CBD metabolites (7-COOH-CBD, 6-OH-CBD, 7-OH-CBD)		x						x
Blood (other) – THC and THC metabolites		x						x
DNA saliva sample^b^		x						

SAFTEE-SI, Systematic Assessment for Treatment Emergent Events – Systematic Inquiry; HDL, high-density lipoprotein; LDL, low-density lipoprotein; HbA1c, glycated haemoglobin; ELFT, electrolyte and liver function tests; NAFLD, non-alcoholic fatty liver disease; FIB-4, fibrosis-4; PANSS, Positive and Negative Syndrome Scale; GAF, Global Assessment of Function; SIMPAQ, Simple Physical Activity Questionnaire; AQoL, Assessment of Quality of Life; CGI, Clinical Global Impression; TOPF, Test of Premorbid Function; BACS, Brief Assessment of Cognition in Schizophrenia; HRSA, Hamilton Rating Scale for Anxiety; CDSS, Calgary Depression Scale for Schizophrenia; TMT, trail making test; FCI, Food Craving Inventory; AUDIT, Alcohol Use Disorders Identification Test; PSQI, Pittsburgh Sleep Quality Index; FBC, full blood count; WCC, white cell count; CBD, cannabidiol; THC, tetrahydrocannabinol.

a. Assessment schedule may vary by plus or minus 5 days for operational convenience.

b. Providing a saliva sample is optional for participants and will be used to explore the presence of known correlates of clozapine and obesity, including variants in genes such as *LEP* and *HTR2C*.

### Efficacy measures

The PANSS positive score will be the primary outcome.^26^ Secondary outcome measures will include the following:

- PANSS total and other subscale scores (negative scale and general psychopathology scale);^26^
- Global Assessment of Function: a numeric scale from 1 to 100 used by clinicians to subjectively rate participants' social, occupational and psychological functioning;^27^
- Simple Physical Activity Questionnaire: a five-item instrument designed to measure physical activity and sedentary behaviour in various populations, including clinical samples with high levels of sedentary behaviour;^28^
- Assessment of Quality of Life 4 Dimensions: a 12-item instrument that captures four dimensions of quality of life, comprising independent living, mental health, relationships and senses;^29^
- Clinical Global Impression: a measure of symptom severity, treatment response and treatment efficacy in treatment studies of people with schizophrenia;^30^
- Test of Premorbid Functioning: a measure of pre-injury IQ and memory ability;^31^
- Brief Assessment of Cognition in Schizophrenia (BACS) Verbal Memory and Learning: a measure of episodic verbal learning memory;^32^
- BACS digit sequencing task: a measure of working memory;^32^
- BACS symbol coding task: a measure of non-verbal functions such as attention, flexibility, speed of processing and abstract reasoning;^32^
- BACS semantic and letter fluency: a verbal fluency test that measures spontaneous production of words that belong to the same category or begin with the same letter;^32^
- Hamilton Rating Scale for Anxiety: a measure of anxiety symptom severity;^33^
- Calgary Depression Scale for Schizophrenia: a nine-item measure of depression in schizophrenia, independent of psychosis symptoms;^34^
- Trail Making Test: a neuropsychological test of visual attention and task switching that can provide information about visual search speed, scanning and speed of processing, mental flexibility, and executive functioning;^35^
- Food Craving Inventory: a 28-item self-reported measure of general and specific food cravings;^36^
- Alcohol Use Disorders Identification Test: a ten-item screening tool to assess alcohol consumption, behaviours, and problems associated with alcohol use;^37^
- Pittsburgh Sleep Quality Index: a 19-item self-reported measure of overall sleep quality assessing seven domains comprising subjective sleep quality, sleep latency, sleep duration, habitual sleep efficiency, sleep disturbances, use of sleeping medication and daytime dysfunction;^38^
- physical health measures including, BMI, waist circumference, waist-to-hip ratio, heart rate, blood pressure, HDL, LDL, triglycerides, total cholesterol, HbA1c, ELFTs, NAFLD and FIB-4.

### Other measures

In addition to the abovementioned measures, we will assess adherence to the trial investigational product. This will be based on self-report and capsule count at each visit. We will perform additional blood tests including a full blood count and measure clozapine/ norclozapine levels, as well as levels of CBD, THC and their metabolites.

Participants will also have the option of providing a saliva sample at baseline. This will be used to explore the presence of known genetic correlates of clozapine and obesity, including variants in genes such as *LEP* and *HTR2C*.^25^ The DNA samples collected in this study will be used to validate associations between DNA single-nucleotide polymorphisms and treatment-resistant clozapine patient populations.

Our research team has used this suite of measures in other clinical trials^39–41^ and found them to be acceptable to study participants with schizophrenia, with low rates of dropout due to burden of clinical measures.

### Statistical analysis

#### Sample size

A power analysis was conducted based on the mean difference in positive PANSS score between treatment arms at the primary analysis endpoint (12 weeks post-baseline assessment). A mean difference in positive PANSS score of 1.30 at 8 weeks^17^ (extrapolated to a conservative difference of −1.8 at 12 weeks, assuming a logarithmic trend over a linear trend), standard deviation difference of 3.739 (Cohen's *d* effect size of 0.48), correlation between assessment timepoints of 0.6, *α* = 0.025 (one-sided), and 1 − *β* = 0.8, with repeated measures (four assessment timepoints) and analysis of variance (ANOVA) as the planned analysis with pairwise contrast, yielded a requirement of 70 participants. Allowing for 20% dropout and rounding up to the nearest multiple of treatment arms (*n* = 2) suggests that a minimum sample of 88 participants is required overall.

#### Data analysis

The effect of CBD relative to placebo on the outcome of 12-week positive PANSS score will be analysed using mixed model repeated measures with fixed effects for treatment arm, time, study site, and treatment arm × time interaction. A random intercept will be included to account for variation in scores between participants. An unstructured variance–covariance structure will be assumed, and where there are convergence issues, a compound symmetry covariance structure will be used instead. Consistent with a treatment policy approach (ICH E9 R1), all participant data will be analysed regardless of missed assessment data, withdrawal from the treatment arm or use of rescue medication (i.e. intention to treat). Suitable contrasts will be used to evaluate the difference in mean total PANSS scores at the primary endpoint (week 12). Missing data will be imputed using multiple imputation by chained equations (MICE) under an assumption of missing at random. A sensitivity analysis will be conducted using a tipping point analysis and the delta adjustment method (e.g. MICE ± 1 PANSS positive score point increments) to evaluate the robustness of the model to the underlying assumptions of missingness. A further sensitivity analysis will be conducted using an alternative analysis of covariance model plus MICE. All other secondary and/or exploratory outcomes will be analysed using the same approach. The number needed to treat will be calculated based on the proportion of participants in each arm who achieve >20% reduction in total PANSS score. Use of co-medications, clozapine/norclozapine levels, and presence of THC and THC metabolites will be considered in the analysis.

#### Interim analysis

Three interim analyses will be performed at 25%, 50% and 75% recruitment, corresponding to *n* = 22, 44 and 66 participants having completed their 12-week assessments. Each interim analysis will provide an ospportunity to evaluate whether the trial can be concluded at an earlier stage, rather than progressing until the target sample has been fully recruited. The interim analysis will evaluate the level of efficacy achieved, conditional on a linear trajectory of change in PANSS positive scores and an end-of-trial Cohen's *d* effect size of 0.48. The upper (early efficacy) and lower (early futility) bounds and their corresponding mean difference at interim analysis are summarised in Table 2. Conditional power at each interim analysis will be evaluated using bootstrap resampling (*n* = 10 000). Dependent on conditional power at interim analyses, we will consider randomisation reallocation to address the level of imbalance to achieve the desired effect size. The interim analysis will also provide an opportunity to reallocate the proportion of participants allocated to placebo and CBD if the study is found to be underpowered. Results of each interim analysis will be non-binding, with results of interim analyses provided to the Data Safety Monitoring Board (DSMB) for further evaluation.

**Table 2:** Summary interim analysis upper and lower bounds

	25% (*n* = 22)	50% (*n* = 44)	75% (*n* = 66)	100% (*n* = 88)
Futility boundary conditional power, 1 − *β_c_*	1 − *β_c_* = 0.99	1 − *β_c_* = 0.93	1 − *β_c_* = 0.86	1 − *β_c_* = 0.80
*δ* at futility boundary	*δ* = −4.74	*δ* = 3.53	*δ* = 6.10	*δ* = 7.76
Efficacy boundary (one-sided)	*P* < 0.0089	*P* < 0.0090	*P* < 0.0092	*P* < 0.0094
*δ* at efficacy boundary	*δ* = 15.64	*δ* = 11.06	*δ* = 8.99	*δ* = 7.76

*β_c_* = probability of type II error at differing time points; *δ,* mean difference achieved at interim analysis.

#### Participant safety

To assess the preliminary safety and tolerability of 1000 mg daily CBD for 12 weeks in people with clozapine-resistant schizophrenia (secondary objective), we will assess:

- the dropout rate between intervention and control groups;
- the numbers of adverse events reported in the intervention and control groups;
- liver function test results at baseline and weeks 6 and 12;
- scores from a structured qualitative interview with participants about their experience with the trial investigational product using the SAFTEE-SI.

All participants will be currently active cases across Queensland Hospital and Health Services. The research team will communicate with clinicians to guarantee that all participants have undergone a regular biannual physical health assessment as part of their standard care. The investigator and clinical trial staff will oversee any adverse events reported during the study. Any adverse events reported between participant consent and final follow-up will be recorded using designated case report forms.

#### Patient withdrawal

All participants have the right to withdraw their consent at any time without prejudice or effect on their ongoing care. This will be clearly discussed during the consent process. If a participant decides to withdraw consent, the trial team will complete a revocation of informed consent form.

#### Reimbursement

Participants will be reimbursed for out-of-pocket expenses, inconvenience and time involved in the trial through the provision of prepaid gift cards. We will provide an AU$20 gift card at the baseline visit and at each fortnightly visit thereafter (total reimbursement AU$140). If the study is terminated by the research team before completion, or a participant withdraws or is withdrawn from the study before completion, a *pro rata* payment will be made at the discretion of the investigator.

### Transparency declaration

D.S. (lead author and manuscript guarantor) affirms that the manuscript is an honest, accurate and transparent account of the study being reported; that no important aspects of the study have been omitted; and that any discrepancies from the study as planned and registered have been explained.

## Discussion

Schizophrenia remains one of the most complex and poorly understood psychiatric disorders. This trial seeks to generate new knowledge to support people with schizophrenia by determining the efficacy of add-on 1000 mg daily CBD for treatment of clozapine-resistant schizophrenia. Existing evidence suggests that CBD is well tolerated in this population with minimal side-effects.^42,43^

This trial has some limitations. First, there is no assurance that the study's findings will be generalisable to all individuals with clozapine-resistant schizophrenia. Although the proposed study is sufficiently powered to detect statistically significant differences between groups, the sample size (*n* = 88) is still small. Moreover, as the study sample is required to fulfil eligibility criteria such as abstinence from substances including amphetamine and cannabis, it may not be entirely representative of the real-world population.

This protocol provides a detailed account of the objectives, design, procedures and analytical approach of this trial. We anticipate that the results of the trial will show significant improvements in participants’ PANSS positive scores and will therefore inform clinical guidance on the maintenance treatment of clozapine-resistant schizophrenia. Moreover, we believe that this trial has the potential to minimise symptom severity and optimise quality of life for individuals with clozapine-resistant schizophrenia.

## Data Availability

The data that support the findings of this study will be available from the corresponding author upon reasonable request.
